# Interactions of Mexiletine with Novel Antiepileptic Drugs in the Maximal Electroshock Test in Mice: An Isobolographic Analysis

**DOI:** 10.1007/s11064-018-2606-8

**Published:** 2018-08-16

**Authors:** Dorota Wróblewska, Monika Rudkowska, Monika Banach, Kinga K. Borowicz-Reutt

**Affiliations:** Independent Unit of Experimental Neuropathophysiology, Department of Pathophysiology, Medical University, Lublin, Poland

**Keywords:** Mexiletine, New generation antiepileptic drugs, Isobolography, Maximal electroshock seizure test, Pharmacodynamic interaction

## Abstract

The aim of the study was to evaluate precisely the type of interactions between mexiletine (an antiarrhythmic drug) and four new generation antiepileptic drugs: lamotrigine, oxcarbazepine, topiramate and pregabalin in the maximal electroshock test in mice (MES). The isobolographic analysis was used to assess the nature of interactions between the tested drugs. Total brain concentrations of antiepileptics were also measured to detect possible pharmacokinetic interactions. The results obtained indicated that the mixture of mexiletine and pregabalin at the fixed ratios of 1:1 and 3:1 led to supra-additive interaction in terms of seizure suppression, while the proportion of 1:3 occurred additive. Synergism was also demonstrated for the combination of mexiletine and topiramate in all three proportions. Combinations of mexiletine with lamotrigine and mexiletine with oxcarbazepine were found to be additive. Adverse-effect profiles of mexiletine, antiepileptics and drug combinations were evaluated in the chimney test (motor coordination) and step-through passive-avoidance task (long-term memory). Mexiletine and drug combinations did not impair long-term memory. Moreover, all combinations of mexiletine with lamotrigine, oxcarbazepine and topiramate had no significant effect on motor coordination. However, the results from the chimney test indicated that pregabalin, administered alone at its ED_50_ dose from the MES-test, significantly impaired motor performance. Similar adverse effects were observed when mexiletine was co-administered with pregabalin at the fixed-dose ratio combinations of 1:1 and 1:3. However, reduction of pregabalin dose at the fixed ratio of 3:1 seems to prevent significant motor impairment. The results may indicate that mexiletine can be considered as an adjunctive drug in antiepileptic treatment, particularly in patients with concomitant cardiac arrhythmia.

## Introduction

Epilepsy is the most common neurological disease affecting approximately 50 million people worldwide. Mortality among patients, particularly those aged 40–50, is two- or threefold higher than in the general population [1]. Unfortunately, despite the introduction of many new antiepileptic drugs, at least one-third of patients suffer from refractory epilepsy and require treatment with more than one antiepileptic drug (polytherapy) to control seizures [2, 3]. However, polytherapy increases the risk of adverse drug reactions, which significantly reduces the patient’s quality of life. Thus, new potential antiepileptic medications should be still searched for. Moreover, the synergistic drug–drug interactions may allow for reduction of drug doses in combined treatment. This in turn can improve undesired effects profile and preserve desired efficacy of duo or polytherapy [4]. Moreover, thanks to the synergistic drug–drug interactions, the drug doses can be reduced in the combined treatment, which is likely to minimize the undesired effects and to maintain the desired efficacy of duo- or polytherapy.

It has been shown that epilepsy and arrhythmia have many common pathophysiological elements, which may also suggest a relationship between antiarrhythmic and antiepileptic drugs. Antiepileptics may exhibit antiarrhythmic activity, although some of them, like phenytoin, carbamazepine and lamotrigine, have also been reported to have arrhythmogenic effects, particularly in overdose. Likewise, certain antiarrhythmic drugs have clear-cut anticonvulsant properties confirmed in numerous studies. However, the biphasic action has also been found, i.e. some antiarrhythmics exhibiting anticonvulsant effects at lower doses may decrease the seizure threshold or even induce seizures at higher doses, e.g. mexiletine [5–12]. Antiarrhythmics have been found to affect not only a seizure threshold but also the action of antiepileptics in different seizure models [13–18].

The probability of interactions between the two groups of medications appears to be considerable since both Class I antiarrhythmics and some anticonvulsants exhibit a similar but not identical mechanism of action based on voltage-gated sodium channel blockade [19, 20]. Such a mechanism can explain the action of mexiletine and antiepileptic drugs on the conductive system of the heart and brain tissue. Moreover, anticonvulsants also act on other channels and/or receptors. Thus, at least additivity (but not indifference) may be expected when a combination of mexiletine with one of antiepileptic drugs is administered. Mexiletine, a local anesthetic and antiarrhythmic drug, belongs to the Class IB of Vaughan Williams system of classification and can be considered an oral analogue of lidocaine. Pharmacological effects of mexiletine are related to blocking the inward sodium current required for the initiation and conduction of impulses. Mexiletine inhibits the fast sodium channel reducing the rate of rise and amplitude of the action potential, which results in increases in the recovery period following repolarization. The drug raises the excitability threshold and decreases automaticity in the His-Purkinje system. Mexiletine has been found to be effective in the treatment of pain and ventricular arrhythmias, such as sustained ventricular tachycardia. The drug has also antiepileptic properties [21–24]. Experimental data have demonstrated that mexiletine showed anticonvulsant effects in mice against seizures induced by electroshock, pentetrazole and a sound signal in audiosusceptible DBA/2 mice [10]. In patients, mexiletine has been shown to be efficacious in symptomatic partial epilepsy, Lennox–Gastaut syndrome and medically refractory early infantile epileptic encephalopathy related to SCN2A mutation [25–28].

The aim of the study was to determine the types of interactions between mexiletine and antiepileptic drugs: lamotrigine, oxcarbazepine, topiramate, and pregabalin in the mouse maximal electroshock (MES) model. It is widely accepted that this experimental animal model of epilepsy reflects tonic–clonic convulsions in humans [29]. Isobolographic analysis was used to characterize the interaction profile and to determine the anticonvulsant effect of drugs. Potential acute adverse effects of mexiletine, antiepileptics and combinations of these drugs were determined in the chimney test (a measure of motor performance impairment) and the step-through passive avoidance task (a measure of long-term memory deficits) in mice. Moreover, brain concentrations of antiepileptics were measured to exclude or confirm possible pharmacokinetic interactions between drugs.

## Materials and Methods

### Animals and Experimental Conditions

Experiments were carried out on adult male Swiss mice weighing 22–25 g. Animals were kept in colony cages with free access to food and tap water and in standardized housing conditions (ambient temperature of 22 ± 1 °C, natural light–dark cycle). After 7 days of acclimatization to laboratory conditions the experiments started. Animals were chosen randomly and assigned to experimental groups comprised of 8–10 mice. All experiments were performed at the same time of a day (between 8.00 a.m. and 3.00 p.m.). Each mouse participated in a particular experiment only once. Additionally, all efforts were made to minimize animal suffering and to use only the number of animals necessary to produce reliable scientific data. The Bioethical Committee of Lublin Medical University approved all experimental procedures of this study, licenses Nos. 29/2014 and 66/2017.

### Drugs

In the study, the following drugs were used: antiarrhythmic drug—mexiletine (Sigma-Aldrich, Slovakia), antiepileptic drugs: lamotrigine (Lamitrin, GlaxoSmithKline, Great Britain), oxcarbazepine (Trileptal, Novartis Pharma, Germany), pregabalin (Lyrica, Pfizer, Great Britain), topiramate (Topamax, Janssen-Cilag, Belgium). Mexiletine was dissolved in distilled water whereas antiepileptic drugs were suspended in 1% solution of Tween 80 (Sigma, St. Louis, MO, USA). Drug solutions were prepared freshly on each day of tests. All examined drugs were administered intraperitoneally in a volume of 10 ml/kg of body weight: mexiletine—15 min, oxcarbazepine—30 min, lamotrigine—60 min, topiramate—60 min, while pregabalin—120 min before the tests.

### Maximal Electroshock Seizure Test

The MES model is one of the most useful tools to determine the anticonvulsant effects of the compounds tested and is considered an animal model of generalized tonic–clonic seizures in humans. In the MES test, electroconvulsions were produced by a Hugo Sachs generator (Rodent Shocker, type 221, Freiburg, Germany). An alternating current (50 Hz, 25 mA, maximum stimulation voltage of 500 V) was delivered with the use of standard auricular electrodes. The electrical stimulus duration was 0.2 s. Tonic hindlimb extension was taken as the endpoint of the test. In the MES test animals received intraperitoneal antiepileptic drugs alone and in combinations with mexiletine. The anticonvulsant effects of the drugs were expressed as their median effective doses (ED_50_s in mg/kg). ED50 values were evaluated for each antiepileptic drug alone and all tested combinations of a given antiepileptic with mexiletine. The median effective dose determines the dose of a drug (or mixture of drugs), which allows to protect half of the animals against MES-induced seizures. Based on the data obtained, a dose–response curve (dose in mg/kg vs. percentage of mice protected) was calculated according to Litchfield and Wilcoxon [30]. This experimental procedure has been fully described by Borowicz et al. [31].

### Chimney Test

Effects of antiepileptics and mexiletine, administered separately and in combinations, on motor impairment were quantified in the chimney test [17, 18, 31, 32]. The animals received mexiletine and antiepileptic drugs at doses corresponding to their ED_50_ values from the MES test. Furthermore, animals were administered combinations of mexiletine with antiepileptic drugs in the ED_50_ dose proportions of 1:1, 1:3, 3:1 previously determined in the MES test and subsequently subjected to the chimney test. In the test, the animals had to climb backwards up the plastic transparent tube (3 cm inner diameter, 25 cm length). Motor impairment was expressed as a percentage of the mice inability to climb backward up within 60 s.

### Step-Through Passive Avoidance Task

The passive avoidance task evaluates the impact of mexiletine, antiepileptic drugs and drug combinations on long-term memory. The experiment requires a two-compartment box: illuminated box (10 × 13 × 15 cm) connected by a sliding door with a large dark box (25 × 20 × 15 cm) equipped with an electric grid floor. On the first day, before the test, each animal received mexiletine or anticonvulsant drug alone at a dose corresponding to ED_50_ value or combination of drugs at their fixed ratios of 1:1, 1:3 or 3:1. The doses of the drugs were identical to those for the MES test. Subsequently, the mice were placed in an illuminated box. Rodents avoid bright places, so after a short time they entered the dark compartment, where they were subjected to a brief aversive stimulus—electric footshock (0.6 mA for 2 s). The mice that did not enter the dark compartment within 60 s were excluded from the test. After 24 h, the same animals were placed again in the illuminated box and observed for up to 180 s. The mice with unimpaired long-term memory did not move to the dark compartment within the observation time [17, 18, 31, 33]. The time after which animals escaped from the illuminated box was noted and the median latencies (retention times) with 25th and 75th percentiles were calculated. Control mice should remember the electrical impulse and stay in the dark compartment for 180 s, therefore, the control retention time fluctuates around the value of 180 (180; 180).

### Measurement of Antiepileptic Drug Concentrations in Brain Homogenates

Total brain concentrations of anticonvulsants were measured to evaluate possible involvement of pharmacokinetic events in the antielectroshock effect of the drug combinations tested. The control groups of mice were treated with an antiepileptic drug and saline. The experimental groups were injected with an antiepileptic and mexiletine. Subsequently, all mice were killed by decapitation at the times chosen to coincide with those scheduled for the MES test. Next, the whole brains were removed from skulls, weighed, and homogenized using Abbott buffer (2:1 v/w) in an Ultra Turax T8 homogenizer (IKA, Staufen, Germany). The brain homogenates were centrifuged at 10,000×*g* for 15 min and the supernatant samples (75 µl) were analyzed with fluorescence polarization immunoassay for lamotrigine, oxcarbazepine or pregabalin content using an Abbott TDx analyzer (Irvine, TX, USA). The antiepileptic drug concentrations were calculated and expressed in micrograms per milliliter of brain supernatants as means ± SD of at least eight determinations.

### Isobolographic Analysis

Isobolographic analysis was used to determine interactions between mexiletine and the antiepileptic drugs studied. This method allows to define precisely the type of pharmacodynamic interactions as synergistic (supra-additive), additive or antagonistic (also termed as sub-additive or infra-additive). Isobolography is based on a statistical comparison of drug doses defined as equieffective. The drugs were administered in different dose combinations and in three proportions (1:1, 1:3, 3:1) of ED_50_ doses of component drugs. The experimental ED_50mix_ and the theoretical additive ED_50add_ values were determined from the dose–response curves of combined drugs according to the methods of Litchfield and Wilcoxon [30] and Tallarida [34]. The 95% confidence limits of ED_50_ values were subsequently transformed to the standard errors of means (SEMs). ED_50mix_ is an experimentally determined total dose of two drugs in the mixture that protects 50% of the animals against MES-induced seizures. The ED_50add_ represents a total additive dose of the drugs in the mixture (calculated from the line of additivity) that theoretically protects 50% of animals against electroconvulsions. Statistical comparison of the experimentally-derived ED_50mix_ values with their corresponding theoretically additive ED_50add_ values was performed using the unpaired Student’s *t* test, according to Porreca et al. [35] and Tallarida [34]. If the experimentally derived ED_50mix_ value is not statistically different from theoretically additive ED_50add_ value, the interaction is regarded as additive. For synergism, the ED_50mix_ is statistically lower than the respective ED_50add_, otherwise, when the ED_50mix_ is statistically greater than the ED_50add_, the interaction is regarded as antagonism. A more detailed description of the isobolographic analysis has been presented in earlier studies [36, 37].

### Statistical Analysis

The ED_50_ values with their respective 95% confidence limits for mexiletine and tested antiepileptic drugs were calculated by computer log-probit analysis according to Litchfield and Wilcoxon [30]. Statistical analysis of drug interactions was performed according to Porreca et al. [35] and Tallarida [34]. The experimental ED_50mix_ values and respective theoretical ED_50add_ values were compared using the unpaired Student’s *t* test. Data from the chimney test were analyzed with the Fisher’s exact probability test, whereas, the results obtained in the step-through passive avoidance task were statistically evaluated using Kruskal–Wallis nonparametric ANOVA followed by post-hoc Dunn’s test. Total brain concentrations of antiepileptic drugs were statistically analyzed using the unpaired Student’s *t* test.

## Results

### Isobolographic Assessment of Interactions between Mexiletine and New Antiepileptic Drugs in Maximal Electroshock-Induced Seizures

**Fig. 1:**
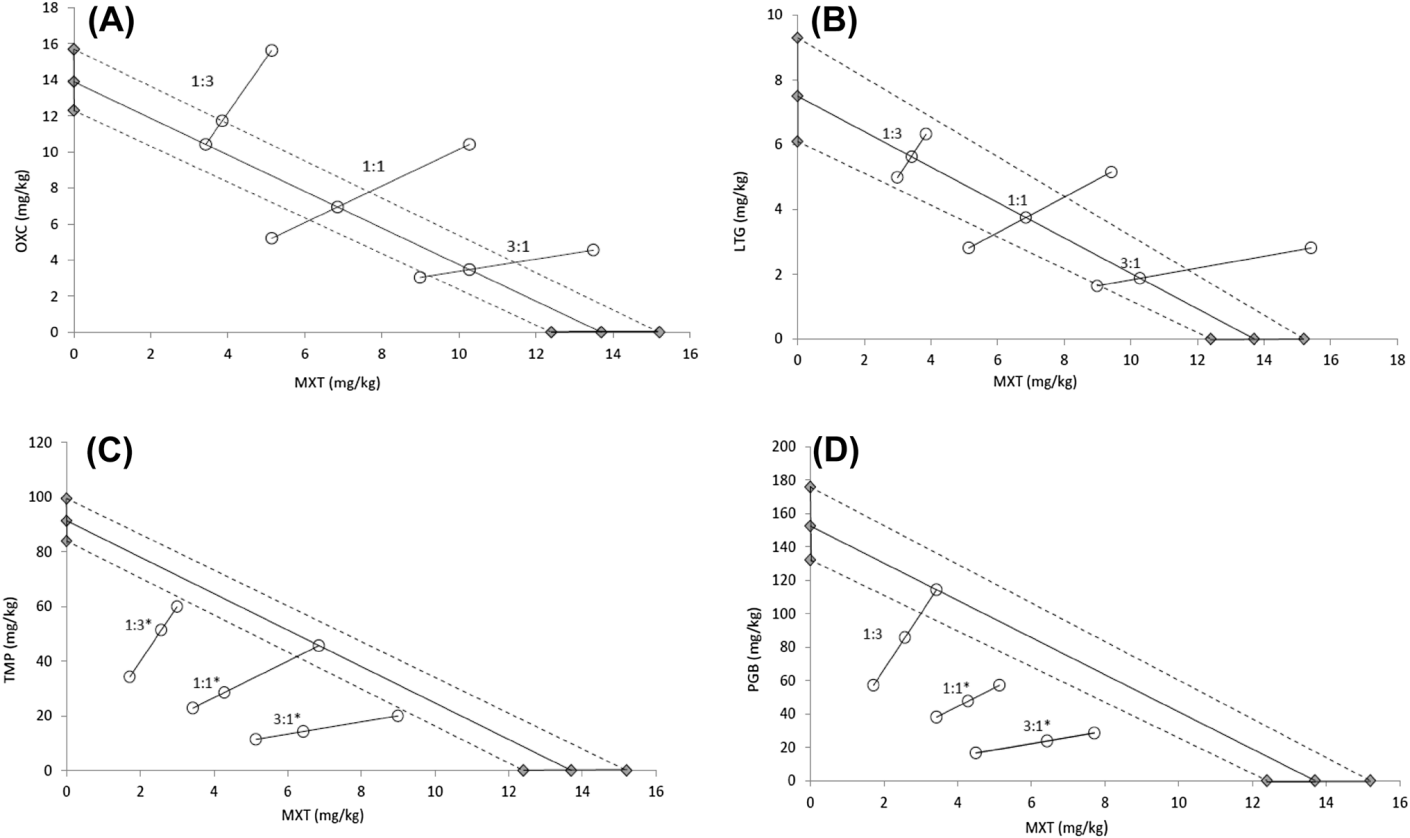
Isobolograms demonstrate interactions between mexiletine and: oxcarbazepine (**a**), lamotrigine (**b**), topiramate (**c**) and pregabalin (**d**) for three fixed-ratio combinations (1:3, 1:1, 3:1) in the maximal electroshock-induced seizures in mice. Median effective dose (ED_50_) for mexiletine is plotted graphically on X-axis, while ED_50_ of the respective antiepileptic drug is placed on Y-axis. The solid line on the X and Y axes represents the 95% confidence limits for the studied drugs administered alone. The straight line connecting both ED_50_ values defines the theoretical line of additivity for a continuum of different fixed-dose ratios. The dotted lines represent the theoretical additive 95% confidence limits of ED_50adds_. The open points (^o^) reflect the experimentally derived ED_50mix_ values (with 95% confidence limits as the error bars) for total dose of drugs mixture expressed as proportions of mexiletine and antiepileptic drug that produced a 50% anticonvulsant effect. The experimental ED_50mix_ values for all fixed-ratios of mexiletine with oxcarbazepine as well as with lamotrigine are close to the line of additivity and thus the observed interactions were additive (**a, b**). The experimental ED_50mix_ values for the mixture of mexiletine with topiramate for all combinations are significantly below the theoretical additive line which indicating synergistic interactions (*p < 0.05; **c**). The mixture of mexiletine with pregabalin for the fixed-dose ratio of 1:3 is near to the line of additivity showing additive interaction, while for the fixed-ratios of 1:1 and 1:3 observed interactions were synergistic (*p < 0.05; **d**). *MXT* mexiletine, *TPM* topiramate, *PGB* pregabalin, *OXC* oxcarbazepine, *LTG* lamotrigine

Isobolographic analysis revealed that there was no statistical difference between the ED_50mix_ and ED_50add_ values for the combinations of mexiletine with oxcarbazepine and mexiletine with lamotrigine and thus, all fixed ratios of these drugs combinations (1:3, 1:1, 3:1) exerted additive interaction in the MES test (Table 1; Fig. 1a, b). The same pattern of interaction was observed between mexiletine and pregabalin at the fixed ratio of 1:3, whereas the remaining combinations with pregabalin (1:1 and 3:1) showed a synergistic character (Table 1; Fig. 1d). Synergism was also demonstrated for the mixture of mexiletine with topiramate at all three fixed-ratio combinations (Table 1; Fig. 1c). The ED_50_ values for mexiletine and antiepileptic drugs obtained from the MES test are presented in Table 2.

**Table 1:** Isobolographic analysis of interactions between mexiletine and pregabalin, topiramate, oxcarbazepine and lamotrigine against MES-induced seizures

Drug combination	F	ED_50add_	ED_50mix_	I
MXT + OXC	1:3	13.9 ± 0.9	15.3 ± 1.0	O
	1:1	13.8 ± 1.5	14.2 ± 1.5	O
	3:1	13.8 ± 1.1	15.0 ± 1.2	O
MXT + LTG	1:3	9.1 ± 0.5	9.1 ± 0.5	O
	1:1	10.6 ± 1.1	11.0 ± 1.12	O
	3:1	12.2 ± 1.0	13.2 ± 1.12	O
MXT + TMP	1:3	71.9 ± 3.1	49.2 ± 3.4*	S
	1:1	52.5 ± 2.3	34.5 ± 3.5*	S
	3:1	33.1 ± 1.5	21.4 ± 1.9*	S
MXT + PGB	1:3	117.7 ± 12.10	84.2 ± 8.7	O
	1:1	83.1 ± 6.5	49.3 ± 3.8*	S
	3:1	48.4 ± 4.6	27.6 ± 2.6*	S

Results are presented as median effective doses (ED_50_ values in mg/kg ± SEM) for two-drug mixtures, protecting 50% of animals tested against MES-induced seizures. ED_50_ values were either experimentally determined from the mixture of two antiepileptic drugs (ED_50mix_) or theoretically calculated from the equation of additivity (ED_50add_). Statistical evaluation of data was performed by using unpaired Student’s *t* test. ED_50add_—theoretically calculated ED_50_; ED_50mix_—experimentally determined ED_50_; I—type of interaction; O—additivity; S—synergism; F—fixed ratio of drug dose combinations (for instance, a fixed-ratio combination of 1:1 was a mixture of equal amounts of mexiletine and antiepileptic drug)

*MXT* mexiletine, *TPM* topiramate, *PGB* pregabalin, *OXC* oxcarbazepine, *LTG* lamotrigine

*p < 0.05 versus the respective ED_50add_ indicating synergistic interaction

**Table 2:** Effects of mexiletine and new antiepileptic drugs against MES-induced seizures

Drug	ED_50_ (mg/kg)	SEM
MXT	13.7 [12.4–15.2]	0.72
OXC	13.9 [12.3–15.7]	0.86
LTG	7.5 [6.1–9.3]	0.80
PGB	152.4 [132.2–175.7]	11.05
TMP	91.3 [83.9–99.4]	3.94

Results are expressed as median effective doses (ED_50_ ± SEM) protecting 50% of animals against MES-induced seizures. All examined drugs were administered intraperitoneally at times corresponding to their maximal antiseizure effect: mexiletine (MXT)—15 min, oxcarbazepine (OXC)—30 min, lamotrigine (LTG)—60 min, topiramate (TPM)—60 min, and pregabalin (PGB)—120 min before the tests.

### Effects of New Antiepileptic Drugs Administered Alone and in Combination with Mexiletine on Long-Term Memory and Motor Coordination

Acute side effects (neurotoxicity) were evaluated for mexiletine, antiepileptic drugs tested and their combinations. Drugs were administered separately at doses corresponding to ED_50_ values from the MES test and in combinations with mexiletine at fixed-dose ratios of 1:1, 1:3, 3:1. The results revealed that pregabalin, administered alone and combinations of mexiletine with pregabalin at the fixed ratio of 1:1 and 1:3 impaired motor coordination in the chimney test. The remaining drugs applied at all fixed ratios had no significant impact on motor coordination. Furthermore, neither mexiletine nor the antiepileptic drugs and the combinations tested affected long-term memory as determined in the passive avoidance task, the median retention times being 180 s (Table 3).

**Table 3:** Effect of mexiletine and new antiepileptic drugs alone and in combination on motor performance and long-term memory

Drug administered (mg/kg)	F	Mice impaired (%)	Median (25, 75) percentiles
Control		0	180 (180; 180)
MXT (13.7)		10	180 (160; 180)
LTG (7.5)		10	180 (180; 180)
OXC (13.9)		0	180 (180; 180)
TMP (91.3)		0	180 (180; 180)
PGB (152.4)		70**	180 (180; 180)
MXT (6.85) + OXC (6.95)	1:1	0	180 (180; 180)
MXT (3.425) + OXC (10.425)	1:3	0	180 (180; 180)
MXT (10.275) + OXC (3.475)	3:1	20	180 (180; 180)
MXT (6.85) + LTG (3.75)	1:1	0	180 (180; 180)
MXT (3.425) + LTG (5.625)	1:3	20	180 (178; 180)
MXT (10.275) + LTG (1.875)	3:1	20	180 (131; 180)
MXT (6.85) + TMP (45.65)	1:1	10	180 (180;180)
MXT (3.425) + TMP (68.475)	1:3	0	180 (180;180)
MXT (10.275) + TMP (22.825)	3:1	0	180 (180; 180)
MXT (4.28) + PGB (47.62)	1:1	70**	180 (180; 180)
MXT (2.57) + PGB (85.725)	1:3	60**	180 (180; 180)
MXT (6.425) + PGB (23.8)	3:1	30	180 (180; 180)

Results are shown as percentage of animals that failed to perform the chimney test and as median retention time (with 25th and 75th percentiles) from the passive avoidance task, assessing long-term memory in mice. Statistical analysis of data from the chimney test was performed by using the Fisher’s exact probability test, whereas the results from the step-trough passive avoidance task were statistically assessed by use of the Kruskal–Wallis ANOVA test followed by Dunn’s post-hoc test

*MXT* mexiletine, *TPM* topiramate, *PGB* pregabalin, *OXC* oxcarbazepine, *LTG* lamotrigine, *F* fixed-dose ratio combination

**p < 0.01 versus control group

### Brain Concentrations of Antiepileptic Drugs

Brain concentrations of antiepileptics were determined in mice that were administered antiepileptic drugs alone or in combinations with mexiletine. When the antiarrhythmic drug was co-administered with pregabalin or oxcarbazepine at the fixed ratio of 1:3 as well as with topiramate in proportion of 1:1, the brain concentrations of antiepileptics were decreased. In contrast, mexiletine combined with pregabalin at the fixed ratio of 1:1 significantly elevated the brain level of the latter drug. Surprisingly, higher doses of mexiletine in the mixtures did not affect total brain antiepileptics concentrations. The results are presented in Table 4.

**Table 4:** Effects of mexiletine on the brain concentrations of new antiepileptic drugs in mice

Treatment (mg/kg)	F	Brain concentration (µg/ml)
TPM (22.83)		2.78 ± 0.62
MXT (10.28) + TMP (22.83)	3:1	2.41 ± 0.67
TPM (45.65)		7.76 ± 2.69
MXT (6.85) + TMP (45.65)	1:1	5.11 ± 1.04**
TPM (68.48)		9.82 ± 1.92
MXT (3.43) + TMP (68.48)	1:3	10.44 ± 1.10
PGB (23.8)		212.46 ± 57.20
MXT (6.43) + PGB (23.8)	3:1	219.25 ± 68.23
PGB (47.62)		372.88 ± 60.77
MXT (4.28) + PGB (47.62)	1:1	519.07 ± 75.29***
PGB (85.73)		787.62 ± 105.65
MXT (2.57) + PGB (85.73)	1:3	508.67 ± 52.37***
OXC (3.48)		0.58 ± 0.038
MXT (10.28) + OXC (3.48)	3:1	0.56 ± 0.033
OXC (6.95)		0.65 ± 0.048
MXT (6.85) + OXC (6.95)	1:1	0.68 ± 0.043
OXC (10.43)		0.79 ± 0.029
MXT (3.43) + OXC (10.43)	1:3	0.67 ± 0.043***
LTG (1.88)		ND
MXT (10.28) + LTG (1.88)	3:1	ND
LTG (3.75)		0.086 ± 0.034
MXT (6.85) + LTG (3.75)	1:1	0.12 ± 0.054
LTG (5.63)		0.36 ± 0.038
MXT (3.43) + LTG (5.63)	1:3	0.37 ± 0.048

Data presented as brain concentrations of antiepileptics (in *µ*g/ml) of eight determinations in mice, and expressed as means ± SD (standard deviation). Statistical analysis of data was performed by use of the unpaired Student’s *t* test

*MXT* mexiletine, *TPM* topiramate, *PGB* pregabalin, *OXC* oxcarbazepine, *LTG* lamotrigine, *F* fixed-dose ratio combination, *ND* not detectable

**p < 0.01; ***p < 0.001 versus an antiepileptic applied alone

## Discussion

The isobolographic analysis showed synergistic interactions between mexiletine and pregabalin (at the dose ratios of 1:1 and 3:1) as well as mexiletine and topiramate (1:3, 1:1; 3:1). The remaining combinations of mexiletine with antiepileptics led to additive interaction (Fig. 1; Table 1). Mexiletine produced no acute adverse effects when combined with lamotrigine, oxcarbazepine and topiramate. Moreover, motor impairment observed in combinations of mexiletine and pregabalin at the dose ratios of 1:3 and 1:1 seems to be related to the effect of pregabalin itself. In proportion 3:1, where the dose of pregabalin was the lowest, no significant motor deficits were detected (Table 3). Interestingly, synergism between mexiletine and topiramate at the dose ratio of 1:1 existed despite a mexiletine-induced decrease in topiramate brain concentration. This may indicate that the pharmacodynamic interaction between the two drugs is strong enough to overcome pharmacodynamic events. A similar situation was observed in the case of the mixture of mexiletine and oxcarbazepine (1:1) or pregabalin (1:3), where additivity was observed despite decreased brain levels of antiepileptics. It seems likely that these unbeneficial pharmacokinetic interactions could mask possible synergy between abovementioned drugs. Surprisingly, pharmacokinetic interactions between mexiletine and pregabalin (were quite different) varied depending on a dose ratio. Mexiletine decreased the brain level of pregabalin at the dose ratio of 1:3, increased it at 1:1, whereas no significant changes were observed in the proportion of 3:1. Therefore, the direction of pharmacokinetic events, at least in mice, should be considered in the context of drug doses.

As already mentioned, mexiletine is defined as a non-selective voltage-gated sodium channel blocker. The mechanism of action of the drug may also explain its anticonvulsant properties. Mexiletine acts by blocking the rapid inward sodium current. Voltage-gated sodium channels play a pivotal role in controlling cellular excitability in the heart muscle and neural tissue and are the molecular targets both for the class I antiarrhythmics and many anticonvulsants. Mexiletine reduces action potential frequency by lengthening the effective refractory period, increase excitability threshold and reduces conduction velocity [38, 39]. The use-dependent sodium channel blocker RS100642s, an analog of mexiletine, revealed anticonvulsant effects in a rat model of transient middle cerebral artery occlusion (MCAo) [40]. Some class I cardiac antiarrhythmics, like: phenytoin, lidocaine and propafenone also demonstrate anticonvulsant properties. Phenytoin belongs to antiepileptic drugs, lidocaine may be useful in the treatment of status epilepticus and refractory epilepsy, while propafenone shows anticonvulsant activity in mice [41–45]. However, all three drugs have been found to induce seizures in overdose (it has been proven that mexiletine, lidocaine and propafenone can induce seizures in overdose). The hyperkinetic myoclonic syndrome has been observed when large doses of mexiletine were administered to mice. In patients, a significant mexiletine overdose resulted in status epilepticus [9–11, 46]. Nevertheless, seizures may reflect toxic effects of different drugs. In contrast, mexiletine applied at therapeutic doses has been effective in refractory epilepsy (particularly symptomatic partial seizures), Lennox–Gastaut syndrome, lidocaine-responsive neonatal epilepsy. A case of two infants with innate refractory seizures, related to mutations in the SCN2A subunit of voltage-gated sodium channels, has also been reported. Although various combinations of antiepileptics remained ineffective, seizures were well controlled by intravenous lidocaine and enteral mexiletine [25–28].

Antiepileptic drugs have numerous molecular targets in the central nervous system; however, the knowledge of all their mechanisms of action seems to be still incomplete. Therefore, to determine many potential interactions between the drugs tested, an experimental evaluation is needed. Lamotrigine and oxcarbazepine act by blocking sodium channels, which results in stabilization of hyperexcited neural membranes and attenuation of sustained high-frequency repetitive firing (SRF). The two drugs have a higher affinity to fast inactivated conformation of sodium channels, they stabilize this inactive form and prevent the return of the channel to the active state. Oxcarbazepine acts primarily through its 10-monohydroxy metabolite (MHD) and anticonvulsant effects of these compounds are probably connected with increased potassium conductance and modulation of high-voltage activated calcium channels. Lamotrigine acts also on other molecular targets. It reduces voltage-dependent calcium currents and inhibits the release of excitatory amino acids, such as glutamate and aspartate [47–49]. Topiramate has a complex mode of action, it antagonizes kainate/AMPA subtype of the glutamate receptors, increases the activity of gamma-aminobutyric acid (GABA), inhibits the carbonic anhydrase enzyme and blocks voltage-dependent sodium and calcium channels, however, in the mechanism different than that presented by classic sodium channel blocking antiepileptics. It has been demonstrated that topiramate decreased the frequency of activation of voltage-sensitive sodium channels and caused a use-dependent, voltage-sensitive and time-dependent limitation of sustained repetitive firing in the cultured mouse spinal cord and neocortical cells [50]. This pattern of activity on the sodium channels is significantly different from that of other antiepileptics, in which there is always a quick limitation or complete blockade of the SRF. Therefore, sodium channel blockade seems not to be the main mechanism, by which topiramate exerts its anticonvulsant effect [51]. Pregabalin acts by binding presynaptically to the alpha2-delta subunit of calcium channels. The drug reduces the calcium release, which in turn inhibits the release of several neurotransmitters in the following order: glutamate, substance P, norepinephrine [52]. According to Deckers et al. [53], if the two drugs applied in combination have different mechanisms of action, synergistic interactions between them are more likely; otherwise additivity probably occurs. Our results seem to confirm this assumption. Synergistic effects occurred in a mixture of drugs with a quite different molecular targets (mexiletine with pregabalin as well as mexiletine with topiramate), while additivity was found between voltage-dependent sodium channels blockers (mexiletine with lamotrigine and mexiletine with oxcarbazepine). In most of the combinations tested in our study, mexiletine did not change the concentrations of antiepileptic drugs in the brain tissue (see Table 4), thus the nature of these interactions can be considered as purely pharmacodynamic. In turn, the synergistic interaction between mexiletine and topiramate in proportion 1:1 occurs despite decreased brain levels of the antiepileptic drug, so it is not due to pharmacokinetic events. Admittedly, synergy between mexiletine and pregabalin in proportion of 1:1 is related to elevated levels of pregabalin, but synergy between the two drugs in proportion of 3:1 is not supported by increased concentrations of the antiepileptic. And again, the pharmacokinetic interaction is not a prerequisite for synergism in this case.

Several reports have revealed that a combination of two sodium blockers seems to be less promising than the mixture of drugs possessing different mechanisms of action. It has been shown that concomitant treatment with classical sodium channel blockers, carbamazepine and phenytoin had no additional medical advantage in mice [54]. Furthermore, according to Łuszczki et al. [55], lamotrigine combined with sodium valproate as well as with topiramate and phenobarbital exhibited synergistic interactions in the MES test in mice, while combinations between lamotrigine and carbamazepine were antagonistic. Another interesting study has demonstrated that the most effective mixture of antiepileptic drugs seems to be a combination of the drug with a single mechanism of action with the drug possessing multiple mechanisms of action. Furthermore, a combination of two voltage-gated sodium channels blockers usually leads to additive interaction in terms of seizure suppression, which is sometimes accompanied by synergistic enhancement of neurotoxic effects [56]. According to Borowicz-Reutt et al. [57], mexiletine interacted additively with phenytoin, carbamazepine and phenobarbital in the MES-induced seizures in mice. In contrast, a combination with valproate resulted in antagonistic interaction, which could be, however, partially due to pharmacokinetic background, since mexiletine significantly lowered the valproate concentration in the brain tissue. Importantly, combinations of this antiarrhythmic drug with classical antiepileptic drugs did not induce significant undesired effects in terms of neurotoxicity. In the present study, a combination of mexiletine with pregabalin resulted in impaired motor coordination in mice; however, this effect seems to be due to the action of pregabalin itself.

Moreover, our results showed that mexiletine interacted pharmacokinetically more frequently with the second-generation antiepileptic drugs, as compared to the first-generation antiepileptics, which contradicts the statement that new generation antiepileptics less often interact pharmacokinetically with other medications [58]. However, pharmacokinetics of antiepileptic drugs in rodents and humans may be different.

Our results demonstrated that, combinations of mexiletine with pregabalin and oxcarbazepine (both at the fixed-ratio of 1:3), as well as with topiramate (1:1) resulted in reducing the concentration of anticonvulsants in the brain. However, despite revealed pharmacokinetic interactions, additivity or even synergism in terms of antiseizure effect was observed. Therefore, pharmacodynamic interactions between these medications seem to prevail over pharmacokinetic events. However, it remains incomprehensible, why mexiletine differently affected brain concentrations of pregabalin depending on drug proportions.

In our study, we evaluated possible effects of pharmacokinetic events on anticonvulsant action of separate drug combinations. Therefore, the brain levels of antiepileptic drugs were measured, starting from the assumption that mexiletine is not a regular antiepileptic and has only possible anticonvulsant effects. Nevertheless, the influence of mexiletine on brain levels of antiepileptics was found to be quite complex and ambiguous. On the other hand, mexiletine itself was active in the MES test. For this reason, we analyzed the available literature data on pharmacokinetics and possible pharmacokinetic interactions between the drugs used in the study. And so, plasma protein binding of mexiletine ranges from 50 to 60%; 85% of the antiarrhythmic is metabolized via CYP2D6 hepatic enzyme and marginally by CYP1A2; 10% of mexiletine is excreted unchanged by kidneys. Pregabalin does not bind to plasma proteins and undergoes negligible hepatic metabolism in humans. 70% of topiramate is excreted unchanged with urine. Its hepatic metabolism is based only on hydroxylation, hydrolysis and glucuronidation processes. 15–41% of topiramate binds to plasma proteins. In turn, lamotrigine is metabolized in the liver by glucuronidation, and its protein binding is 55%. Finally, oxcarbazepine is metabolized to its pharmacologically active 10-monohydroxy metabolite (MHD) by cytosolic enzymes. MHD is metabolized further by conjugation with glucuronic acid. Protein binding of MHD is 40%. None of the four drugs affect the action of P-glycoprotein. There are no data on possible interactions between mexiletine and pregabalin, topiramate, lamotrigine or oxcarbazepine [58–64].

This knowledge, however, does not bring us closer to explaining such complex pharmacokinetic interaction between mexiletine and second-generation antiepileptics. Nevertheless, it should be remembered that pharmacokinetics of drugs in mice and humans may be not the same.

Summing up, the antiarrhytmic may be useful as a adjunctive medication in combination with not only classical but also new generation anticonvulsants. It should be underlined that favorable interactions between drugs may lead to reduction of doses and thus adverse effects induced by anticonvulsants without losing their activity. Further research is needed to evaluate more precisely possible mechanisms of pharmacodynamic or pharmacokinetic interactions between mexiletine and antiepileptics.

## Conclusions

The obtained results indicate that mexiletine shows its own anticonvulsant activity and may potentiate the action of some second-generation antiepileptic drugs against mouse MES-induced seizure. In our opinion, mexiletine deserves more attention from both a preclinical and clinical point of view. Confirmation of experimental results in clinical conditions may contribute to the development of rational polytherapy of epilepsy.
